# The fate and effect of monensin during anaerobic digestion of dairy manure under mesophilic conditions

**DOI:** 10.1371/journal.pone.0192080

**Published:** 2018-02-08

**Authors:** Osman A. Arikan, Walter Mulbry, Clifford Rice, Stephanie Lansing

**Affiliations:** 1 Sustainable Agricultural Systems Laboratory, USDA-ARS, Beltsville Agricultural Research Center, Beltsville, Maryland, United States of America; 2 Department of Environmental Science and Technology, University of Maryland, College Park, Maryland, United States of America; 3 Department of Environmental Engineering, Istanbul Technical University, Istanbul, Turkey; Purdue University, UNITED STATES

## Abstract

There is growing concern about residual antibiotics and feed additives in the manure of treated animals because of the effects of these residues in the environment. Monensin is the most widely used ionophore coccidiostat in the U.S. The objective of this study was to determine the fate and effect of monensin during the anaerobic digestion of dairy manure. Duplicate plug flow field-scale digesters were operated using non-amended dairy manure and dairy manure amended with monensin to 1 and 10 mg/L for 56 days at 30°C at an organic loading rate of 1.4 kg VS/m^3^-d and 17-day hydraulic retention time. Results showed that monensin was reduced approximately 70% during anaerobic digestion. Methane production from digesters using manure amended with 1 mg/L monensin was comparable to that from digesters operated without added monensin. However, digesters using manure amended with 10 mg/L monensin yielded 75% less methane than digesters using manure without added monensin. These results suggest that anaerobic digestion is an effective treatment for reducing, but not eliminating, monensin in dairy manure. Monensin did not reduce methane production at concentrations expected in dairy manure at recommended dosage rates.

## Introduction

Monensin is a polyether ionophore commonly used in livestock animals, including cattle, swine, and poultry, as a coccidiostat and for growth promotion [1–4]. It is the most widely used ionophore in the United States (U.S.) [5] and is one of the few feed additives permitted by the U.S. Food and Drug Administration to increase milk production for lactating dairy cows [6]. An estimated 1500 tons of monensin (600 tons for cattle and 900 tons for poultry) were used for non-therapeutic purposes in the U.S. in 2001 [1]. Extensive use of monensin for dairy cows was also documented in other countries, including Australia, Argentina, Brazil, Canada, New Zealand, and South Africa [7]. Donoho et al. [8] reported that approximately 50% of monensin administered to cattle is excreted in manure as a parent compound.

There is growing concern about residual antibiotics and feed additives, such as monensin, present in the manure of treated animals because of the effects of these residues in the environment. The widespread use and persistence of monensin have led to its detection in manure (0.3–4.5 mg/L) [9, 10], soil (0.0004 μg/kg) [4], surface water (0.01–0.05 μg/L) [11, 12], groundwater (0.04–0.39 μg/L) [13] and sediment (1.5–31.5 μg/kg) [12]. Monensin was the most frequently (63%) detected antibiotic in irrigation return flows of an intensively managed agricultural watershed in south-central Idaho [14]. The EC_50_ of monensin in the aquatic environment is highly species dependent, ranging from 0.2 to 7.3 mg/L [15]. Monensin is classified as a high priority compound based on its high usage and high toxicity profile [16].

Anaerobic digestion is an established and proven technology for the treatment of animal manure [17] and is one possible means of reducing the amount of monensin that is ultimately released into the environment [18]. Significant reduction of antibiotic residues during anaerobic digestion can be achieved but it is compound specific [18, 19]. Although the effect of monensin on anaerobic digestion has been studied in laboratory scale systems [10, 20–22] there are no comparable studies using field-scale (FS) systems. In addition, there is very limited information on the fate of monensin during anaerobic digestion of dairy manure. Residues of feed additives in manure can have negative effects on treatment systems such as nitrifying systems [23] and anaerobic digesters [19, 24] and can cause methanogenic consortia inhibition [25].

The objective of this study was to determine the fate and effect of monensin on anaerobic digestion using replicate, field-scale dairy manure digesters. Duplicate plug flow field-scale digesters were operated using non-amended dairy manure and dairy manure amended with monensin to 1 and 10 mg/L. Monensin concentrations in digester influents and effluents were monitored to calculate monensin removal rates. Methane (CH_4_) production and digester effluent characteristics (pH, alkalinity, COD, volatile fatty acids) were monitored to evaluate the effect of monensin on digester efficiency and stability.

Based on results from laboratory-scale studies [21, 22], we predicted that extractable levels of monensin would decrease from 10 to 40% during digestion and that the decrease would be independent of the initial monensin concentration. Based on results from other laboratory-scale studies [9,10], we predicted that methane production would be partially inhibited (10 to 45% inhibition) and significantly inhibited (55 to 100% inhibition) in digesters containing 1 and 10 mg/L monensin, respectively.

## Materials and methods

### Chemicals

**Fig 1:**
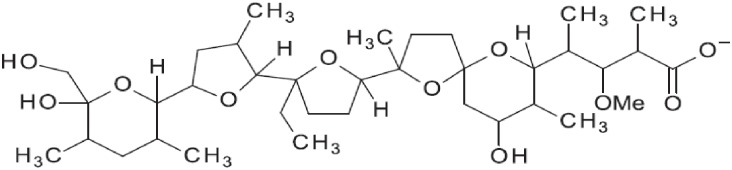
Chemical structure of monensin.

Monensin as a sodium salt (90–95% purity) was purchased from Sigma-Aldrich (St. Louis, MO, USA). The structure of monensin is shown in Fig 1. HPLC grade acetonitrile and methanol were purchased from Fisher Scientific (Pittsburg, PA, USA). All other reagents used in this study were analytical grade. Water used for analysis was purified by using reverse osmosis and activated carbon. Stock solution of monensin (100 mg/L in acetonitrile) was prepared monthly and stored at -20°C in the dark. Working solutions (0.001, 0.01, 0.1, 0.5 and 1.0 mg/L) were prepared weekly by diluting the stock solution with acetonitrile and stored at 4°C in the dark.

### Substrate

Dairy manure was obtained from the USDA’s Dairy Research Unit at the Beltsville Agricultural Research Center (BARC) (Beltsville, MD, USA) (39.0319° N, 76.8913° W). The dairy's manure treatment system has been described previously [26]. Table 1 shows characteristics of the solid-separated dairy manure used as feedstock in this study. Lactating cows at the BARC dairy routinely received monensin in their diet corresponding to a daily dose of 325 to 350 mg per cow. Consequently, the solids-separated dairy manure used in the study contained 0.2 mg/L monensin (Table 1).

**Table 1:** Characteristics of dairy manure used as feedstock in the study.

Parameter	Concentration^1^
Monensin, mg/L	0.21 ± 0.03
Total solids (TS), g/L	31.9 ± 2.0
Volatile solids (VS), g/L	23.8 ± 1.8
pH	7.1 ± 0.3
Alkalinity, g/L as CaCO_3_	6.9 ± 0.3
Total COD, g/L	37.7 ± 4.0
Soluble COD, g/L	9.4 ± 1.0
Total Kjeldahl Nitrogen (TKN), g/L	2.2 ± 0.3
Total Phosphorus (TP), g/L	0.22 ± 0.02

^1^ Values are means ± std. dev. from duplicate samples.

### Field-scale anaerobic digestion

Anaerobic digestion experiments were carried out using six modified Taiwanese-model field-scale (FS) digesters at the BARC dairy [26]. Each FS digester has a total capacity of 3 m^3^ and a working volume of 2 m^3^. The FS digesters are plug-flow reactors, which are the most common systems in the U.S. [27], operated without mixing.

Prior to the study, each FS digester was filled with 1 m^3^ of inoculum from the BARC digester and 1 m^3^ of dairy manure. Each digester was subsequently loaded with dairy manure and maintained at 30 ± 2°C since our previous study showed that CH_4_ production was not significantly different between digesters operated at 30 and 35°C [28]. After steady state conditions were achieved, duplicate FS digesters were operated for additional 56 days using dairy manure (non-amended) and dairy manure amended with monensin to 1 and 10 mg/L. The digesters were loaded five days a week with 160 L of dairy manure, corresponding to a hydraulic retention time (HRT) of 17 days. The mean organic loading rate (OLR) was 1.4 ± 0.1 kg VS/m^3^-d during the study. This value is within the range of suggested OLR values (1–3 kg VS/m^3^-d) for animal manure digestion [29]. After feeding was completed each day, the influent kettle and manure pipes lines were rinsed with water to minimize carryover of monensin. Steady state conditions, defined as stable CH_4_ production, CH_4_ content, soluble chemical oxygen demand (COD) and volatile fatty acid (VFA) values, were reached in the digesters after they were operated for a minimum of two HRT.

Digesters were emptied at the end of the study. Multiple samples were collected from each digester as it was emptied in order to determine the amount of monensin that had settled with manure solids in the bottom of the digesters. A mass balance of monensin was calculated for each digester using the total mass of monensin fed to individual digesters over the 56-day period, the total mass of monensin in the effluent, and the quantity that stayed in the digester within the settled solids.

### Amendment of manure with monensin

One pair of digesters was operated using non-amended manure containing 0.2 mg/L monensin because of existing monensin use at the dairy (described in section 2.2 above) Additional pairs of digesters were operated using manure amended with two monensin levels (1 and 10 mg/L), corresponding to final monensin levels of 1.2 and 10.2 mg/L in the amended manures, respectively. These two amendment levels were chosen as they spanned the range of values expected using the minimum (115 mg/cow/day) and maximum (600 mg/cow/day) dosage rates [30], 50% and 85% excretion rates, and a daily liquid manure rate of 50 L per cow [21].

Dry mix feed grade Rumensin 90 (containing 20% monensin) (Elanco Animal Health, Greenfield, IN) was used for monensin addition. The concentration of monensin was verified after extraction and analysis using the method below. Rumensin 90 was added into the influent kettle while the kettle was being filled with dairy manure. The kettle was stirred continuously to provide better mixing of monensin with dairy manure.

### Analytical methods

Monitoring of digester temperatures, biogas production, and determination of methane concentrations has been described previously [25]. Influent and digester effluent samples were collected weekly for determination of monensin, pH, alkalinity, soluble and total chemical oxygen demand (COD) according to Standard Methods [31]. Determination of volatile fatty acids (VFA) (acetic, propionic, n-butyric, and n-valeric acids), total Kjeldahl nitrogen (TKN), total phosphorus (TP), total solids (TS) and volatile solids (VS) values of samples were determined as described previously [25].

### Extraction of monensin

Manure samples were extracted for analysis of monensin using a slight modification of the method described by Varel et al. [10]. Briefly, 10 mL dairy manure samples were extracted with 20 mL of acetonitrile followed by an extraction with 20 mL of an acetonitrile: ethyl acetate solution (50:50 v:v). After each solvent addition, samples were mixed for 1 minute using a vortex mixer, followed by centrifugation (5 min, 1200 X g). Extracts from the second solvent addition (acetonitrile: ethyl acetate) were sonicated for 10 minutes in a sonication bath (Elma, E100H, Elmasonic, Germany) prior to centrifugation. Following centrifugation, the upper extraction layers were decanted into 250 mL glass beakers and allowed to evaporate in a fume hood at room temperature (approximately two days). Dried samples were dissolved in 40 mL of methanol and aliquots of this material were transferred to amber autosampler vials prior to analysis of monensin by liquid chromatography mass spectrometry (LC/MS/MS).

### LC/MS/MS analysis for monensin

Monensin concentrations were determined using a Waters 2695 LC and Micromass Quattro Ultima MS (Waters Corp., Milford, MA, USA) with an electrospray source using an XBridge C_18_ column (150 mm x 2.1 mm i.d., 5 μm) (Waters Corp., Milford, MA, USA) in conjunction with an XBridge C_18_ guard column at 60°C. Positive ionization modes were used for detection. The injection volume was 5 μL. A gradient separation was utilized involving a mixture of solvent A (0.1% formic acid:acetonitrile, 50: 50, v/v), solvent B (100% water), solvent C (100% methanol), and solvent D (100% acetonitrile). The solvent gradient program was as follows: 0–2 min. 100% B, flow rate (FR) 0.2 mL/min; 8–20 min. a linear gradient to 100% A, FR 0.4 mL/min; 20–23.5 min. 20% A, 80% D, FR 0.4 mL/min; 23.5–27 min. 100% C, FR 0.3 mL/min; 27–32 min. 100% D, FR 0.3 mL/min; and 32–33 min. returning to the starting condition. The column was stabilized for 7 minutes with 100% B prior to injection of the next sample. The total run time was 40 minutes. Analytes were detected using multiple reaction monitoring methods available on the triple quadrupole mass spectrometer. The parent and daughter ions used for monensin identification and quantitation were 693.7 and 479.8 Da, respectively. Analyte concentrations were calculated by the external standard method using five-point standard calibration curves (fits with > 0.9 r^2^ values). Peak integration and quantitation were performed automatically using the MassLynx 4.0 software (Waters Corp., Milford, MA, USA).

### Statistical analyses

Statistical significance between results from digesters amended with monensin and non-amended digesters were evaluated. Differences in CH_4_ production and content, total and soluble COD, pH, and alkalinity at the steady state were statistically analyzed separately. One-way ANOVA and Tukey’s test were used for comparing the effects of different treatments. Differences were considered significant for p-values less than 0.05. All statistical analyses were performed using R statistical software [32].

## Results and discussion

### Determination of extraction efficiencies for monensin

To determine extraction efficiencies, duplicate samples of influent and effluent manure were spiked with monensin at 0.1, 0.5, 1, 5 and 10 mg/L and extracted as described above. Recovery results shown in Table 2 were calculated as means of duplicate samples at each concentration and corrected for background monensin levels. Average recovery values of monensin from influent and digester effluents were 78 ± 9% and 90 ± 3%, respectively. Recovery values in both matrices appeared to be independent of initial concentration. These values are comparable to the recovery value of 79% reported by Varel et al. [10] using cattle manure slurry.

**Table 2:** Recovery of monensin in dairy manure.

Matrix	Recovery (%)^1^ Spike level (mg/L)					Average
	0.1	0.5	1.0	5.0	10.0	
Influent manure	86 ± 12	74 ± 13	74 ± 9	79 ± 8	75 ± 1	78 ± 9
Effluent manure	98 ± 5	93 ± 2	90 ± 2	82 ± 7	86 ± 1	90 ± 3

^1^ Values are means ± std. dev. from duplicate samples.

### The fate of monensin during anaerobic digestion

**Fig 2:**
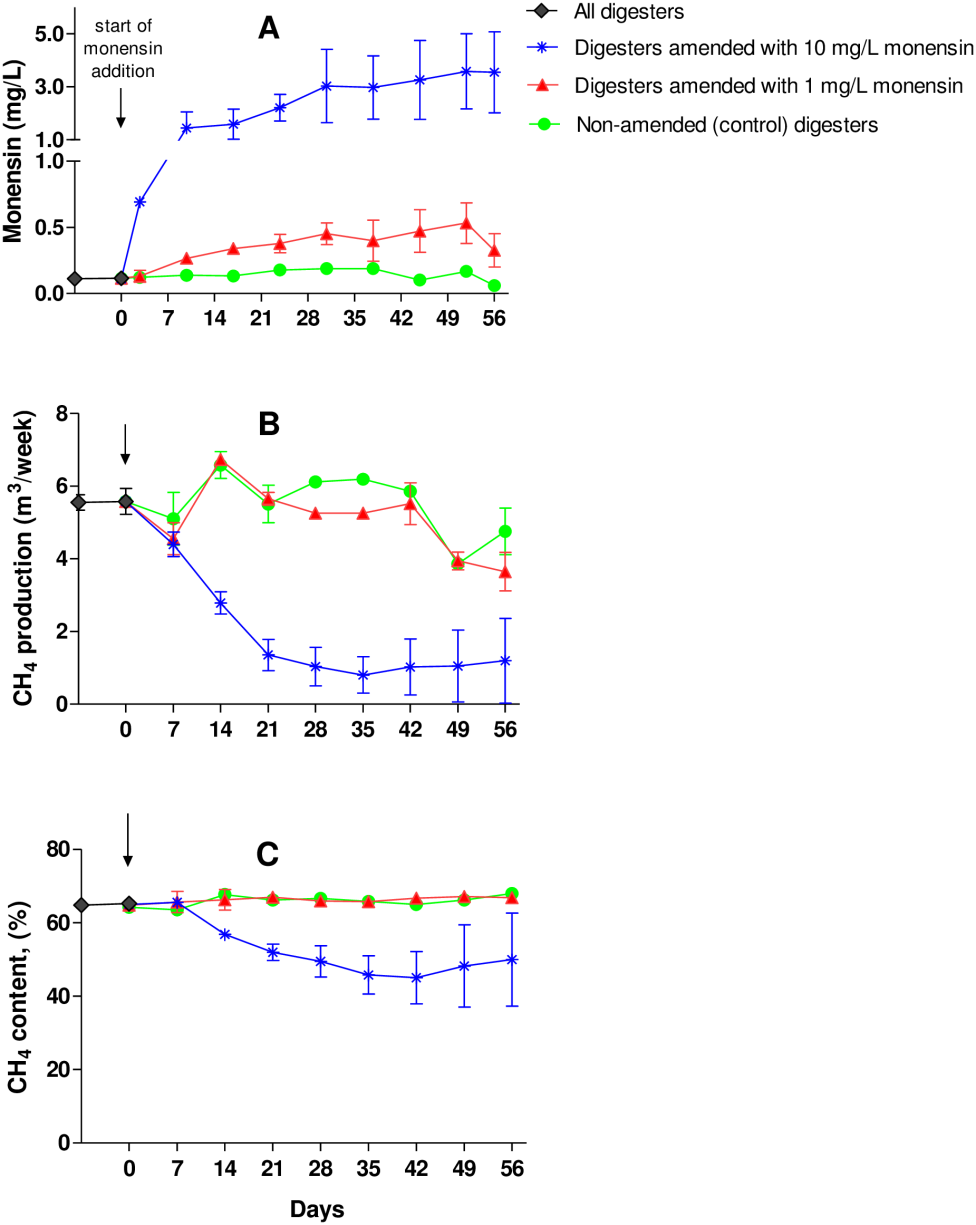
Effluent monensin concentrations (mg /L) (a); weekly CH_4_ production (m^3^/week) (b); and CH_4_ content (%) (c) from the field-scale (FS) digesters operated using dairy manure (non-amended) and dairy manure amended with monensin to 1 and 10 mg/L. Values are the means from duplicate digesters. Standard deviations are shown as error bars.

Our results show that effluent monensin levels under steady-state conditions were approximately 70% lower than influent levels. Effluent monensin levels from digesters containing monensin amended manure increased gradually after addition of monensin (Fig 2a). Steady-state mean effluent monensin concentrations were 0.14± 0.06, 0.44 ± 0.1 and 3.28 ± 1.1 mg/L from the digesters containing non-amended manure, and manure amended with 1 and 10 mg/L monensin, respectively (Table 3). Mass balance results of monensin show that roughly 20% of input monensin was due to settling of insoluble monensin residues in the bottom of these unmixed digesters (Table 4). Since solids settling is common in these unmixed plug flow digesters [33], accumulation of monensin in the digesters was not surprising.

**Table 3:** Steady state results of field-scale (FS) digesters operated using dairy manure (non-amended) and dairy manure amended with monensin to 1 and 10 mg/L (mean ± std. dev.).

Parameter	Non-amended digesters	Digesters amended with 1 mg/L monensin	Digesters amended with 10 mg/L monensin
Effluent Monensin ^1^, mg/L	0.14 ± 0.06	0.44 ± 0.1	3.28 ± 1.1
Weekly biogas production, m^3^/week	8.1 ± 1.5	7.1 ± 1.3 (0.23) ^2^	2.0 ± 1.0 (<0.001)
Weekly CH_4_ production, m^3^/week	5.4 ± 1.0	4.7 ± 0.9 (0.23)	1.0 ± 0.6 (<0.001)
CH_4_ content, %	66.3 ± 1.2	66.6 ± 0.7 (0.99)	47.3 ± 7.5 (<0.001)
Specific CH_4_ production, m^3^/kg-VS	0.22 ± 0.05	0.19 ± 0.04 (0.23)	0.04 ± 0.02 (<0.001)
Effluent soluble COD, g/L	2.5 ± 0.3	2.7 ± 0.2 (0.39)	10.0 ± 0.6 (<0.001)
Effluent total COD, g/L	23.5 ± 5.7	22.6 ± 2.4 (0.87)	30.3 ± 2.1 (<0.001)
Effluent alkalinity, g/L as CaCO_3_	9.8 ± 0.8	9.8 ± 0.6 (0.99)	7.5 ± 0.3 (<0.001)
Effluent pH	7.6 ± 0.1	7.6 ± 0.1 (0.83)	7.0 ± 0.1 (<0.001)
Effluent acetic acid, mg/L	105 ± 30	220 ± 95 (0.31)	4087 ± 283 (<0.001)
Effluent propionic acid, mg/L as acetic acid	6 ± 5	9 ± 7 (0.99)	424 ± 95 (<0.001)
Effluent butyric acid, mg/L as acetic acid	< 0.1	< 0.1 (0.99)	114 ± 25 (<0.001)
Effluent valeric acid, mg/L as acetic acid	< 0.1	< 0.1 (0.99)	8 ±3 (<0.001)

^1^ Effluent monensin concentrations in the digesters spiked with 1 and 10 mg/L were not corrected for background monensin levels.

^2^ Values in parenthesis show p-values between monensin amended digesters and non-amended digesters.

**Table 4:** Mass balance of monensin (mean ± std. dev.) in digesters containing monensin-amended manure.

	Digesters amended with 1 mg/L monensin	Digesters amended with 10 mg/L monensin
Monensin input, %	100	100
Monensin in the effluent, % of input	31 ± 8	23 ± 9
Monensin reduction, % of input	69 ± 8	77 ± 9
Monensin in settled solids, % of input	16 ± 4	23 ± 13

Our experiments do not resolve whether the balance of monensin reduction is caused by degradation, mineralization or binding of monensin to the organic matrix. The literature describing the fate of monensin during anaerobic digestion is very limited and there is no information about the mechanism of monensin degradation under anaerobic conditions. Early metabolic studies using [^14^C]-labeled monensin provide the most information with regard to aerobic degradation pathways but offer few clues about possible anaerobic degradation routes [8,9]. These studies showed that there were over 50 metabolites from cattle and rats but that no single metabolite was dominant [8]. Metabolites were primarily the results of O-demethylation and hydroxylation at different positions along the carbon backbone rather than from conjugation or fragmentation of the molecule. Kennedy et al. [22] was suggested that acidogenic bacteria may partially degrade monensin. Our removal values are comparable to those from incubation using fresh (and presumably anaerobic) feces from monensin-fed cattle [9]. Results showed that monensin levels decreased 42% (from 4.5 to 2.6 mg/L) over 10 weeks at 37°C. More recently, Varel et al. [10] reported much lower values of monensin reduction (8 and 27% reduction of monensin from an initial level of 0.3 mg/L at 38 and 55°C, respectively) during a 4-week batch experiment using medicated calf manure. Although we do not know why our results differ from that study, it is possible that differences in the microbial consortia or solid-content of the respective manures may play a role.

### Effect of monensin on anaerobic digestion

Our results show that addition of monensin at the low rate did not have a significant effect on methane production, but that methane production decreased 75% in digesters containing manure amended with monensin at the high rate. Prior to the addition of monensin, weekly CH_4_ production and CH_4_ content values were not significantly different (p > 0.05) among the six digesters. The average weekly CH_4_ production and CH_4_ content values from the digesters were 5.4 ± 0.4 m^3^/week and 64.7 ± 0.8%, respectively (Fig 2b and 2c). The average specific CH_4_ production value of the digesters was 0.24 ± 0.02 m^3^/kg-VS. This value is within the range of the CH_4_ productivity values (0.14–0.34 m^3^/kg-VS) reported for separated dairy manure at mesophilic temperatures [28, 34–36]. Under steady state conditions after monensin addition, CH_4_ production and content values from digesters containing 1 mg/L monensin amended and non-amended manure were not significantly different (Fig 2b and 2c, Table 3). However, 10 mg/L monensin amended digesters and non-amended digesters were significantly different with respect to CH_4_ production and content (Fig 2b and 2c, Table 3). The average steady state weekly CH_4_ production values of the digesters fed with non-amended manure and manure amended with 1 and 10 mg/L monensin were 5.4 ± 1.0, 4.7 ± 0.9 and 1.0 ± 0.6 m^3^/week, respectively (Fig 2b, Table 3). Digesters operated with manure amended with 1 and 10 mg/L monensin produced 12% and 75% less CH_4_, respectively, compared to non-amended digesters. The average CH_4_ content values in the biogas from non-amended, 1 and 10 mg/L monensin amended digesters were 66.3 ± 1.2, 66.6 ± 0.7 and 47.3 ± 7.5%, respectively (Fig 2c, Table 3).

**Fig 3:**
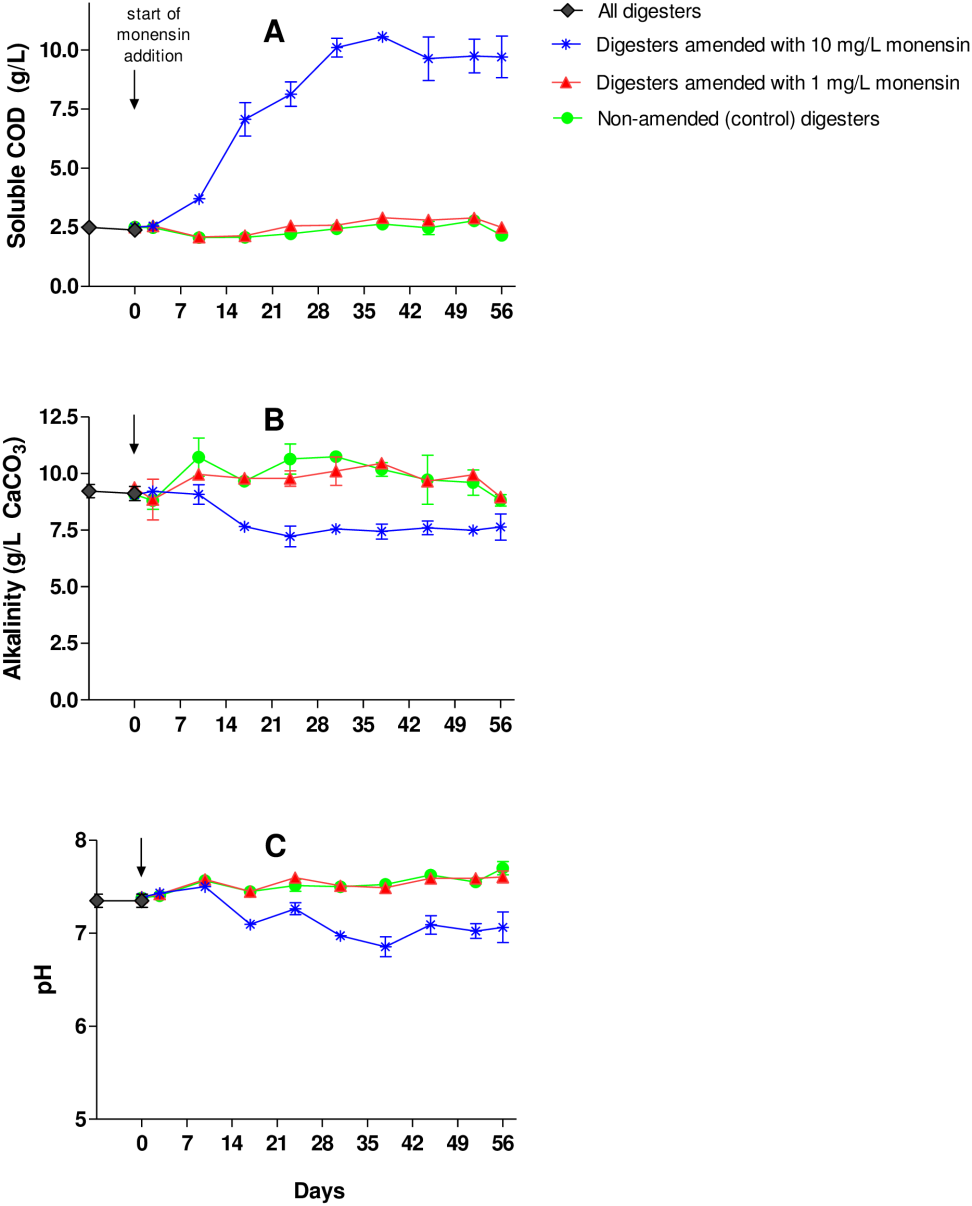
Effluent soluble COD concentrations (g/L) (a); effluent alkalinity, (g/L as CaCO_3_) (b); and effluent pH (c) from the field-scale (FS) digesters operated using dairy manure (non-amended) and dairy manure amended with monensin to 1 and 10 mg/L. Values are the means from duplicate digesters. Standard deviations are shown as error bars.

Parameters affecting digester stability (soluble COD, pH, alkalinity,VFA) were significantly affected by monensin amendment at the high rate but not at the low rate (Fig 3, Table 3). After the addition of the manure containing 10 mg/L monensin, effluent soluble COD values gradually increased, while pH and alkalinity values decreased (Fig 3). The 1 and 10 mg/L monensin amendments resulted in 10 and 300% increases in effluent soluble COD at steady state, respectively, compared to digesters containing non-amended manure (Fig 3a, Table 3). Alkalinity concentrations in digesters containing manure amended with 10 mg/L monensin were 23% lower than those from digesters containing non-amended and manure amended with 1 mg/L monensin (Fig 3b, Table 3). The lowered buffering capacity was likely due to lower ammonia concentrations. Ammonia levels of digesters amended with 10 mg/L (848 ± 32 mg/L) monensin were approximately 14% lower than 1 mg/L monensin amended and non-amended digesters (979 ± 10 mg/L and 963 ± 18 mg/L, respectively). Although digester pH was not affected at 1 mg/L, at 10 mg/L monensin the pH decreased from 7.6 to 7.0, with the pH remaining above 7 due to the strong buffering capacity (Fig 3c, Table 3). Monensin addition at 1 mg/L resulted in a two-fold increase in acetic acid concentrations (220 ± 95 mg/L) compared to non-amended (105 ± 30 mg/L), but the values were not significantly different (Table 3). However, 10 mg/L monensin increased acetic acid levels approximately 40-fold (4087 ± 283 mg/L) compared to non-amended and resulted in accumulation of propionic and butyric acids (Table 3).

Our field-scale results regarding the effect of monensin on methane production during anaerobic digestion are in broad agreement with results from previous lab-scale studies. Our results closely match those of Kennedy et al. [22] who found a 10% reduction and complete suppression in biogas production at 1.0 and 8.0 mg/L monensin doses, respectively, for continuous systems in 22 L digesters operated at 35°C. Varel and Hashimoto [20] found a complete inhibition of CH_4_ production when manure from cattle fed with monensin was added to 4 L semi-continuous digesters at 35 or 55°C. However, the monensin inhibition decreased after a six-month adaptation period. Monensin levels in the influent manure were not determined in that study. The high sensitivity of methanogens towards monensin was demonstrated in several studies by Hilpert and coworkers [21,37]. Wildenauer et al. [38] determined that there was 45% less CH_4_ for monensin concentrations of 2 and 5 mg/L, and 55% less CH_4_ for 10 mg/L compared to the control in 1.5 L semi-continuous digesters using cattle manure. They suggested that monensin inhibits fermentative, acetogenic, and methanogenic bacteria in a nonselective manner. Wedegaertner and Johnson [39] showed that ruminal CH_4_ production decreased 26% by monensin treatment. Whetstone et al. [40] showed that monensin inhibits the degradation of protein by rumen microbes in vitro.

## Conclusions

Our study showed that approximately 70% removal of monensin was achieved by anaerobic digestion of dairy manure at 30°C using field-scale digesters. About 20% of this reduction was due to monensin associated with solids that settled in the digesters. Our results also showed that while a concentration of 1 mg/L monensin did not have significant effect on CH_4_ production and process stability, a concentration of 10 mg/L monensin significantly reduced CH_4_ production and resulted in digester instability.
